# Transcriptional Regulatory Network for the Development of Innate Lymphoid Cells

**DOI:** 10.1155/2015/264502

**Published:** 2015-08-26

**Authors:** Chao Zhong, Jinfang Zhu

**Affiliations:** Molecular and Cellular Immunoregulation Unit, Laboratory of Immunology, National Institute of Allergy and Infectious Diseases, National Institutes of Health, Bethesda, MD 20892, USA

## Abstract

Recent studies on innate lymphoid cells (ILCs) have expanded our knowledge about the innate arm of the immune system. Helper-like ILCs share both the “innate” feature of conventional natural killer (cNK) cells and the “helper” feature of CD4^+^ T helper (Th) cells. With this combination, helper-like ILCs are capable of initiating early immune responses similar to cNK cells, but via secretion of a set of effector cytokines similar to those produced by Th cells. Although many studies have revealed the functional similarity between helper-like ILCs and Th cells, some aspects of ILCs including the development of this lineage remain elusive. It is intriguing that the majority of transcription factors involved in multiple stages of T cell development, differentiation, and function also play critical roles during ILC development. Regulators such as Id2, GATA-3, Nfil3, TOX, and TCF-1 are expressed and function at various stages of ILC development. In this review, we will summarize the expression and functions of these transcription factors shared by ILCs and Th cells. We will also propose a complex transcriptional regulatory network for the lineage commitment of ILCs.

## 1. Introduction

The mammalian immune system is composed of innate and adaptive arms. T cells and B cells derived from the lymphoid lineage belong to the adaptive immune system. CD4^+^ T cells perform a “helper” function via production of effector cytokines after differentiation and activation. In comparison to the “cytotoxic” feature of both adaptive CD8^+^ T cells and innate cNK cells, the “helper” feature of CD4^+^ Th cells was considered to be a unique characteristic of the adaptive system acquired during evolution. However, over the past few years several groundbreaking works on a novel member of the innate immune system, the innate lymphoid cell (ILC) [1], have dramatically changed our knowledge about the composition of the innate lymphoid lineage [2] and led us to reconsider the relationship between innate and adaptive lymphoid lineages in the context of evolution.

Like other lymphocytes, ILCs also develop from the common lymphoid progenitors (CLPs) found in fetal liver and adult bone marrow. They were not discovered and classified as a new lymphocyte family until recently, partly due to their distinct enrichment in nonlymphoid tissues such as mucosal tissues, skin, and adipose tissues, with scarce distribution in lymphoid tissues. Their lack of any known lineage surface markers may also contribute to their belated discovery. In actuality, scientists noticed certain subsets of ILCs such as lymphoid tissue inducers (LTis) as early as the 1990s [3, 4], but it was not until three independent reports on type 2 cytokine producing innate lymphoid cells (ILC2s) in 2010 [5–7] that people began to recognize the possible existence of an innate population with a “helper” feature mirroring adaptive Th cells. The nomenclature of innate lymphoid cells (ILCs) was then formed based on the existence of “helper” lymphocyte in the innate arm of the immune system [1].

Similar to the classification of Th cells, mature helper-like ILCs can be categorized into three groups based on their master regulator expression and signature effector cytokine production. ILC2s, the innate counterpart of Th2 cells, express high levels of GATA-3 and are capable of producing type 2 cytokines such as IL-5 and IL-13 [5–7]. ILC3s express ROR*γ*t and are capable of producing IL-22 and IL-17, similar to Th17/Th22 cells [8–10]. ILC3s can be divided into a CCR6^+^ lineage and a CCR6^−^ lineage [11]. The CCR6^+^ lineage includes lymphoid tissue inducer (LTi) cells, while CCR6^−^ILC3 can give rise to a special population of ROR*γ*t^+^ ILC3s that express the natural cytotoxicity receptor (NCR) NKp46 (encoded by* Ncr1*) in mice and NKp44 (encoded by* Ncr2*) in humans. These cells are referred to as NCR^+^ ILC3s. CCR6^−^ILC3s can also express T-bet which drives further development of this lineage into the NCR^+^ stage [11–13]. Finally, ILC1s are the innate counterpart of Th1 cells. They are T-bet positive and better IFN-*γ* producers than cNK cells [14, 15]. Absence of ROR*γ*t and Eomes expression in ILC1s distinguishes them from ROR*γ*t-expressing NCR^+^ ILC3 and Eomes-expressing cNK cells.

Similar to the relationship between ILCs and Th cells, “cytotoxic” cNK cells represent the innate counterpart of the adaptive cytotoxic CD8^+^ T cells [16]. Accordingly, both cNK and CD8^+^ T cells express the transcription factor Eomes. Initially, the nomenclature of ILCs included cNK cells, a notion many scientists still hold. But in this review, we limit ILCs to helper-like ILCs to distinguish these special innate lymphocytes from cNK cells.

## 2. Functions of ILCs

A defining function of ILCs is their production of a similar set of effector cytokines as those produced by CD4^+^ Th cells in the adaptive immune system. This feature of ILCs enables them to mount robust immune responses at the innate stage via acting on other immune or structure cells. Through production of IL-5 and IL-13, ILC2s may induce the first wave of eosinophil recruitment and stimulate epithelial and smooth muscle cells, during type 2 responses to helminth infection or allergen inoculation [17, 18]. Steady state production of IL-22 by ILC3s is crucial for the homeostasis between host and commensals within the mucosal tissues [19]. Upon infection, ILC3s are also the major innate source of IL-22 after receiving IL-23 stimulation [8, 10]. ILC1 cells are relatively scarce in the gut but enriched in the liver in steady state [14, 20]. During early type 1 responses, ILC1s are better IFN-*γ* producers than cNK cells and they provide the initial protection in mice infected with* T. gondii* [14]. Interestingly, ILC1s are also much more potent TNF*α* producers compared to cNK cells.

Some ILCs can directly interact with Th cells. ILC2s and Th2 cells may collaborate to mount robust type 2 immune responses during the effector phase. Some ILC2s express MHC class II and thus are able to stimulate Th2 cells to produce IL-2, which in turn promotes ILC2 proliferation and cytokine production [21, 22]. Some ILC3s, mainly within the CCR6^+^ lineage, also express MHC class II [23]. MHCII^+^ ILC3s can directly present antigen peptide to CD4^+^ T cells. However, possibly due to the lack or low level of costimulatory molecules CD80/CD86 on ILC3s, this type of antigen presentation functions through a suppressive mechanism to maintain the homeostasis of commensal specific Th cell in the colon [24].

ILCs have additional functions, which may or may not be shared by Th cells. For example, ILC2 can produce amphiregulin, which facilitates the repair and reorganization of damaged tissues after viral infection [25]. ILCs are also involved in regulating metabolism. For example, ILC2s are enriched in adipose tissues and contribute to the beiging of white adipose tissue through production of cytokines and/or methionine-enkephalin [26, 27]. Through IL-22 and lymphotoxin production, ILC3s can induce intestinal epithelial cell expression of fucosyltransferase 2 (Fut2) and thus regulate the epithelial fucosylation, which provides the metabolic substrates for commensals [28, 29].

## 3. Specific Regulators for Distinct ILC Subsets

The development, maturation, and maintenance of distinct ILC subsets are regulated by a set of specific transcriptional regulators, including T-bet, GATA-3, and ROR*γ*t, similar to the regulation of effector Th cell differentiation. A master regulator that determines Th cell differentiation towards a particular subset also seems to direct the development of the related ILC subset. In addition, other factors such as ROR*α*, Bcl11b, and Ahr are involved in regulating the development and functions of ILC subsets.

### 3.1. T-bet, GATA-3, and ROR*γ*t

Mirroring their critical functions during Th1, Th2, and Th17 differentiation, the master regulators T-bet, GATA-3, and ROR*γ*t are also implicated in fate determination of ILC subsets. GATA-3 is expressed at much higher levels in ILC2s compared with other ILC subsets.* Gata3* deficiency during any stages of ILC2 development will eliminate this lineage. Furthermore, GATA-3 is required for the maintenance and function of fully developed ILC2s [30, 31]. Deletion of* Gata3* in mature ILC2s results in dramatic diminution of IL-5 and IL-13 production followed by the rapid disappearance of these cells. GATA-3 is also expressed in ILC1 and ILC3 cells as well as in ILC progenitors, in which GATA-3 has a critical function; we will discuss GATA-3 function during early ILC development in detail in the progenitor section below.

ROR*γ*t is uniquely expressed by ILC3s in both CCR6^+^ and CCR6^−^ subsets. ROR*γ*t deficiency results in a complete loss of ILC3s but not ILC1s or ILC2s. Some CCR6^−^ILC3s also express T-bet [11]. T-bet induction in ILC3s may be driven by Notch signals [12], IL-23 stimulation, and the microbiota [11]. The gradient increment of T-bet levels directs further development of CCR6^−^ILC3s into NCR^+^ ILC3s. Some cells may even turn off ROR*γ*t expression to become T-bet^+^NCR^hi^ ex-ILC3s. In addition, T-bet regulates IFN-*γ* production by the NCR^+^ ILC3s [11].

Besides CCR6^−^ILC3s, ILC1s also express T-bet. Accordingly, T-bet deficient mice lack ILC1s and NCR^+^ ILC3s but have normal ILC2s and CCR6^+^ ILC3s. ILC1s were previously confused with cNK cells as both express T-bet and are capable of producing IFN-*γ*. However, cNK cells express Eomes while ILC1s do not. Therefore, Eomes is a good marker to distinguish cNK cells from ILC1s. In addition, ILC1s may also express cell surface markers such as CD49a [14], CD160 [15], and/or CD127, which are usually absent on cNK cells.

### 3.2. ROR*α*

ROR*α* is highly expressed by ILC2s and is necessary for their development [32, 33]. ROR*α* deficiency results in dramatic reduction of ILC2s but does not affect the development of other ILC subsets. The mechanism of how ROR*α* regulates the development of ILC2s is still not clear. ROR*α* deficiency does not seem to affect Th2 cell differentiation and function. Thus, mice reconstituted with ROR*α* deficient bone marrow are useful tools for studying ILC2 functions during immune responses.

### 3.3. Bcl11b

Bcl11b is a critical factor during early stages of T cell development [34, 35]. But during ILC development,* Bcl11b* deficiency only blocks the development of ILC2s as shown by two independent studies published this year [36–38]. Bcl11b is expressed as early as the common ILC progenitor stage and it may suppress the development of ILC3s. The mechanism of how Bcl11b specifies ILC2 fate is yet to be determined. Unlike GATA-3 function in mature ILC2s, Bcl11b deletion does not affect the maintenance or function of mature ILC2s despite Bcl11b being highly expressed in mature ILC2s.

### 3.4. Ahr

The aryl hydrocarbon receptor (Ahr) is well known to be involved in Th17 cell differentiation and function [39]. Ahr is also expressed by both CCR6^+^ and CCR6^−^ ILC3s [40, 41]. In adult* Ahr* deficient mice, ILC3 cell number in the gut is dramatically reduced, probably due to the defective accumulation and/or enhanced apoptosis of ILC3s. Ahr is also critical for the function of ILC3s by regulating IL-22 production and the Ahr effects on ILCs seem to be ILC3 specific.

## 4. Progenitors for ILCs

ILCs, cNK cells, and T cells all develop from CLPs found in fetal liver and adult bone marrow. At late stages of T cell development, naïve CD4^+^ and CD8^+^ T cells develop from the CD4^+^CD8^+^ double positive cells in the thymus whereas CD4^+^ T effector Th cells are differentiated from naïve CD4^+^ T cells in the periphery. Considering the functional similarity between mature ILCs and CD4^+^ Th cells, it is reasonable to propose a similar developmental pathway shared by these cells. Thus, a hypothesis concerning ILC development is that, like T cells, there may be a common progenitor for all innate lymphocytes, including ILCs and cNK cells, after the CLP stage. In a subsequent stage, a common ILC progenitor would be capable of giving rise to all ILCs, in parallel with the potential of naïve CD4^+^ T cells to become different Th effector cells. This hypothesis is supported by the fact that certain gene deletions affect the development of all innate lymphocytes and/or all ILCs. Indeed, during the last year, we have witnessed a few breakthrough studies in identifying the common progenitors for ILCs [14, 42].

### 4.1. Id2^+^ ChILP

Id2 is required for the development of all ILC populations since its deficiency results in the loss of all ILCs. Using an Id2 reporter mouse strain, several groups have identified and confirmed a common ILC progenitor. A Lineage^−^CD127^+^Flt-3^−^Integrin*α*_4_ *β*_7_^+^ population has been reported in a stage after CLP and has lost the potential to become T, B, and cNK cells [43]. Id2 expression within this population has been shown by expression of an Id2 fluorescent reporter. Previously reported immature ILC2s in bone marrow are also Id2^+^; however, they can be excluded by CD25 staining. Transfer of these Id2^+^CD25^−^ progenitor cells gives rise to all “helper” ILCs. The multipotential capacity of these Id2^+^CD25^−^ progenitors for all ILC subsets was also confirmed by* in vitro* single cell development assay. These Lineage^−^CD127^+^Flt-3^−^Integrin*α*_4_ *β*_7_^+^Id2^+^CD25^−^ progenitors are thus termed as “common helper-like innate lymphoid progenitors” (ChILPs).

### 4.2. GATA-3 Function in Common ILC Progenitors

In addition to the critical role in maintenance and function of ILC2s, GATA-3 is also crucial in the general development of all ILCs. It has been recently reported that* Gata3* deficiency prior to the CLP stage affects the development of all ILCs in a cell intrinsic manner, indicating that GATA-3 is a critical regulator for ILC development [31]. In mice carrying a Cre construct driven by the* Vav1* promoter to conditionally delete* Gata3* at the hematopoietic stem cell stage, dramatic defects in all IL-7R*α*-expressing ILCs in the periphery are noted. Moreover, these* Gata3* conditional-deficient mice do not generate lymph nodes or Peyer's patches, consistent with the finding that the development of functional LTi cells is defective at the fetal stage. They also succumb to* Citrobacter rodentium* infection, consistent with another report showing that GATA-3 mediates the development of ILC3s by using chimera mice with hematopoietic precursor cells from E12.5–13.5* Gata3* ^−/−^ embryos [44]. The essential role of GATA-3 during ILC development is consistent with its indispensable function during CD4^+^ T cell development [45] after the CD4^+^CD8^+^ stage in the thymus. Consistent with its critical role during ILC development, GATA-3 expression levels in the common ILC progenitors, such as ChILPs, are comparable to that in ILC2s [14]. Thus, GATA-3 is likely a master regulator for the development of common ILC progenitors.

Given that both Id2 and GATA-3 are highly expressed in ILC progenitors, it is reasonable to speculate that while Id2 may direct the acquisition of the “innate” feature of ILCs similar to cNK cells GATA-3 may play an indispensable role in directing the “helper” feature of ILCs similar to CD4^+^ T cells. Because of this, GATA-3 may distinguish the “helper” lineage from “cytotoxic” cNK lineage during innate cell development. Altogether, Id2 and GATA-3 coexpression during the common ILC progenitor stage may establish a special regulatory network that determines the “innate” and “helper” features of the ILCs.

### 4.3. PLZF^+^ Common ILC Progenitor

By analyzing a PLZF fate mapping mouse strain, researchers found that the majority of mature ILCs have expressed PLZF with the exception of the CCR6^+^ ILC3 lineage [42]. PLZF is hardly detectable in mature ILCs but is transiently expressed by a subset of the ChILP population in fetal liver and adult bone marrow [14]. These PLZF^+^ progenitors are multipotential cells and are able to give rise to all ILCs except CCR6^+^ LTi cells. Furthermore, these PLZF^+^ progenitors do not develop into T cells, B cells, or cNK cells. PLZF^+^ progenitors may develop after the ChILP stage and have lost the potential to develop into LTi lineage. The function of PLZF during ILC development remains elusive particularly because PLZF is only transiently expressed during the early development stage. Furthermore, although* Zbtb16* ^−/−^ mice with PLZF deficiency have altered ILC development especially for ILC2s, all the ILCs can still be detected in these mice [42].

An obvious issue for these defined common ILC progenitors is that they are actually heterogeneous populations. Within the ChILP cells, there are PLZF^+^ and PLZF^−^ progenitors. There should be some CCR6^+^ ILC3 progenitors within the PLZF^−^ population, which will never turn on PLZF expression. Even within the PLZF^+^ population, only a minority of the cells have the potential to become multiple ILCs during* in vitro* single cell development assay. It is likely that both Id2^+^ ChILPs and PLZF^+^ common ILC progenitors contain a large fraction of partially committed ILC immediate progenitors. The identification of an “authentic” common progenitor multipotent for all ILCs is still required. Meanwhile, in addition to fetal liver and adult bone marrow, the common ILC progenitors may also exist in other tissues, such as fetal intestine, as was shown in a recent study using arginase-1 reporter mice [46]. This report suggests that we should not limit ourselves to study fetal liver and adult bone marrow cells to gain further knowledge on ILC progenitors.

### 4.4. Immediate ILC Subset Precursors

As mentioned above, many ILC progenitors found in fetal liver and adult bone marrow may have already committed partially to a specific ILC lineage. Indeed, Bcl11b-expressing progenitors appear within the ChILP population; yet these cells have already committed to the ILC2 fate [37]. There are also abundant immature ILC2s, presumably the most immediate ILC2 precursors, present in the bone marrow; these cells express mature ILC2 markers Sca-1 and CD25 [30]. Immature ILC1s also exist in bone marrow termed as Eomes^−^CD49a^+^NK1.1^+^NKp46^+^ [14]. PLZF fate mapping analysis indicates that most immature ILC1s have expressed PLZF [47] and thus are derived from the PLZF^+^ ILC progenitors rather than the cNK progenitors.

### 4.5. Common Progenitors for ILCs and cNK Cells

A couple of other transcription factors, Nfil3 [48–51] and TOX [52], have also been found to be involved in the development of both ILCs and cNK cells. Since their expression is detected earlier than Id2 expression, which occurs at the ChILP stage, the expression of these transcription factors may mark even earlier common progenitors for both ILCs and cNK cells, similar to CD4^+^CD8^+^ thymocytes during T cell development. However, the phenotypes of ILC development in mice deficient in either of these regulators are not as dramatic as those resulting from* Id2* or* Gata3* deficiency. The mechanisms through which Nfil3 and TOX function during ILC and cNK cell development require further investigation.

**Figure 1:**
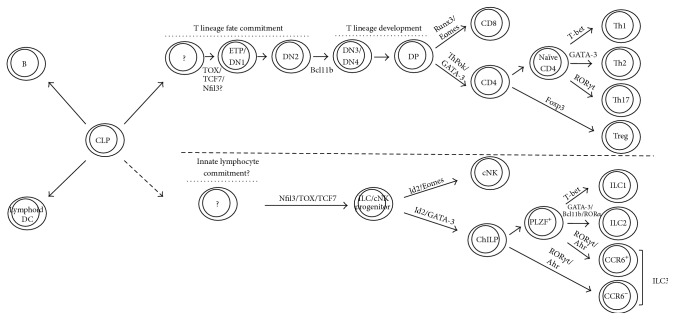
Parallels between the development of innate lymphocytes and T cells. Lymphoid DCs, B cells, T cells, and innate lymphocytes including ILCs and cNK cells are derived from common lymphoid progenitors (CLPs). While innate lymphocytes mainly develop in the bone marrow, T cells develop in the thymus. T cell development passes through a fate determination (from ETP/DN1 to DN2 to DN3) stage before CD4^+^ and CD8^+^ T cells develop separately from progenitor-like CD4^+^CD8^+^ DP cells. After CD4^+^ T cell development, effector Th cells are differentiated from naïve CD4^+^ T cells upon activation. In parallel, for the innate lymphocyte development, there may also be a stage when the innate fate is determined followed by the generation of ILC and cNK common progenitors. ILC and cNK lineage then develop separately under the guidance of distinct master regulators. Similar to effector Th cells differentiating from naïve CD4^+^ T cells, all mature ILC subsets also develop from a common progenitor. Therefore, ILCs share with T cells in utilizing multiple transcription factors at similar stages during their development/differentiation.

Given the functional similarities between the innate ILCs and cNK cells and the adaptive CD4 and CD8 T cells, it is reasonable to propose that ILCs and cNK cells follow a similar developmental pathway to that of T cells. A CLP may give rise to a common progenitor for all innate lymphocytes including ILCs and cNK cells, which subsequently gives rise to ChILPs. Distinct ILC subsets are further developed from the PLZF^+^ ChILP stage as the differentiation of Th effectors from naïve CD4^+^ T cells. Thus, the development of innate lymphocyte subsets, including ILCs and cNK cells, to a certain extent, mirrors that of T cells (Figure 1).

## 5. Regulatory Network in Common ILC Progenitor

As discussed above, more and more regulators are being found to be involved in the development of ILCs. Interestingly, most of these regulators are also involved with T cell development. However, distinct from T cell development, the regulatory functions of the various transcription factors during ILC development are not related to TCR-mediated thymic selection, which, to some extent, may explain why the deficiency of certain regulators has distinct effects on ILC and T cell development. Although deletion of either one of the newly identified genes, including* Nfil3*,* TOX,* or* Tcf7* [53, 54], results in defective ILC development to a various extent, none of these defects are as severe as that resulting from* Id2* or* Gata3* deficiency. Thus, it is possible that in the early progenitors of the ILC lineage, these regulators may function through a network designed for fine-tuning ILC development. Id2 and GATA-3 might form the core complex, while other regulators are involved in tuning the optimal function of the core components.* Id2* or* Gata3* deficiency dramatically affects ILC development, whereas deletion of other regulatory components results in various incomplete defects of ILC development.

### 5.1. Nfil3

Nuclear factor interleukin-3 (Nfil3; also known as E4-binding protein 4, E4BP4), a basic region leucine zipper transcription factor, is critical for cNK cell development [55]. However,* Nfil3* deficiency also affects ILC development [48–51]. Based on this, it is possible that Nfil3 is expressed and functions at a progenitor stage common to both ILCs and cNK cells.* Nfil3* deficient mice showed dramatic defects during the transition from CLP to ChILP stage [48, 51]. However, ILC development is not completely abolished in* Nfil3* deficient mice; in the periphery, there are still substantial numbers of ILCs that have escaped the developmental block, although the population is too small to control infectious challenges. Nfil3 expression is induced by IL-7 signaling, which is also critical for the development of ILCs. Nfil3 may work by regulating TOX [50] and Id2 [51] expression. After development, Nfil3 maintains a very high level of expression in mature ILCs, a level higher than that in cNK cells. Nfil3 is dispensable for ILC3 maintenance; however, its function in other mature ILCs remains unclear.

### 5.2. TOX

Thymocyte selection-associated high-mobility group box protein (TOX) is a member of the high-mobility group box superfamily. It contains a DNA-binding domain and is required for the development of T [56, 57], NK [58], NKT [59], and LTi cells [58]. A new study this year showed that TOX also has broad effects in ILC development [52]. In* Tox* deficient mice, the ChILPs as well as mature ILCs were severely reduced compared with these cells in wild type mice. There is an intrinsic defect in the Notch signaling pathway in* Tox* deficient ChILPs [52], which may explain the defect in ILC development in* Tox* deficient mice.

### 5.3. TCF-1

T cell factor 1 (TCF-1) is a critical transcription factor for T cell fate specification at the early development stage [60].* Tcf7* (gene encode TCF-1) deficiency affects both ILCs [53, 54] and cNK cells [61]. Similar as during T cell development, TCF-1 is regulated by Notch signaling and may be involved in the induction of IL-7R and GATA-3 expression during ILC development [53, 54].

### 5.4. Putative Regulatory Network in ILC Progenitors

**Figure 2:**
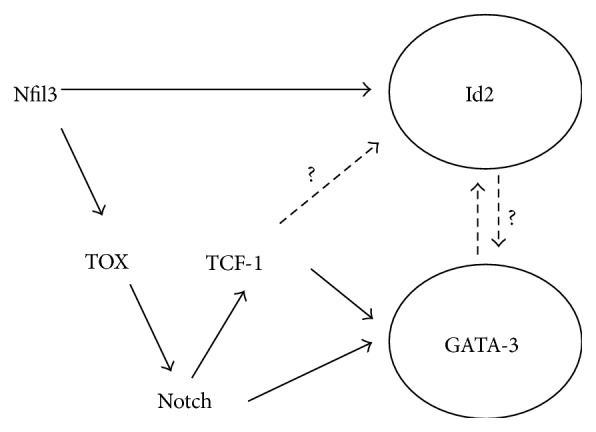
A potential transcriptional regulatory network determining the generation of a common ILC progenitor. Critical transcription factors during common ILC progenitor development are expressed at various stages. After the CLP stage, Nfil3 expression increases followed by Id2 and TOX induction. TOX then induces Notch, which is required for TCF-1 and GATA-3 upregulation. TCF-1 further enhances GATA-3 expression. Although it has not been reported, TCF-1 may also play a role in enhancing Id2 expression. The regulatory network eventually results in optimal Id2 and GATA-3 expression in the common ILC progenitors. Once Id2 and GATA-3 expression reaches a threshold, they orchestrate the acquisition of the “innate” and “helper” features of the ILCs, possibly also with assistance from other transcription factors.

Based on the sequential expression of Nfil3, TOX, TCF-1, Id2, and GATA-3 and the knowledge we have concerning their relationships in different systems, it is likely that Nfil3 expression initially increases after the CLP stage, which in turn regulates the expression of TOX and Id2. After Id2 is turned on, the core assembly involved in directing the “innate” features of ILC development begins to function. Although GATA-3 is expressed at low levels at the CLP stage, its function during ILC development requires high expression levels. TOX is involved in the regulation of Notch signaling pathway, which in turn regulates TCF-1 and GATA-3. TCF-1 is then required for optimal expression of GATA-3. Upon increased expression of GATA-3, the “helper” transcription factor assembly would execute the gene expression program needed for ILC function. In this way, Nfil3, TOX, and TCF-1, in connection with the Notch pathways, form a network to prepare for the upregulation of Id2 and GATA-3, which together form the executive regulatory network to direct lineage fate determination of ILCs, possibly with continued assistance from the initiation transcription factors such as Tcf7, Tox, and Nfil3 (Figure 2). Additional experiments are required to confirm this proposed regulatory network model and it is likely that additional regulators for ILC development may soon be discovered.

## 6. Conclusions

Studies on the newly identified ILCs in recent years have enriched our knowledge on the innate lymphoid lineage development and have provided us with more evidence supporting the close relationship between the innate and adaptive immune systems during evolution. The classification of the ILC population was initially based on their capacity to produce a similar set of effector cytokines compared to Th cells. Subsequent studies have revealed that the development of ILCs is also regulated by a similar set of key transcription factors required for T cell development. After the CLP stage, innate lymphoid lineages and T cells lineages start to develop separately but the regulatory mechanisms may still be shared. Nfil3, TOX, and TCF-1 expression at an early stage immediately after CLP in the innate lymphoid lineage may mark the common progenitor for both ILCs and cNK cells. ILC and cNK lineages are further separated by the induced expression of Id2 and GATA-3 in the common ILC progenitors. At later stages, additional regulators are upregulated to drive the distinct fates of different ILC subsets. In conclusion, the ILC cell fate is precisely regulated during development by a network of multiple serially expressed regulators, which may form a regulatory network during development and render the developed ILCs with both “innate” and “helper” features.
